# Epidemiology of congenital Chagas disease 6 years after implementation of a public health surveillance system, Catalonia, 2010 to 2015

**DOI:** 10.2807/1560-7917.ES.2019.24.26.19-00011

**Published:** 2019-06-27

**Authors:** Luca Basile, Pilar Ciruela, Ana Requena-Méndez, Mª José Vidal, Eva Dopico, Andrea Martín-Nalda, Elena Sulleiro, Joaquim Gascon, Mireia Jané, the Working Group of Congenital Chagas disease in Catalonia

**Affiliations:** 1 Public Health Agency of Catalonia, Barcelona, Spain; 2 CIBER Epidemiology and Public Health CIBERESP, Carlos III Health Institute, Madrid, Spain; 3 Barcelona Institute for Global Health (ISGlobal), Hospital Clínic- Universitat de Barcelona, Barcelona, Spain; 4 Laboratori Clínic de l'Hospitalet, Hospitalet de Llobregat, Barcelona, Spain; 5 Pediatric Infectious Diseases and Immunodeficiencies Unit, Hospital Universitari Vall d'Hebron (HUVH), Vall d'Hebron Research Institute (VHIR), Universitat Autònoma de Barcelona (UAB), Barcelona, Spain; 6 Microbiology Department, Hospital Universitari Vall d'Hebron (HUVH), PROSICS Barcelona, Universitat Autònoma de Barcelona (UAB), Barcelona, Spain; 7 The members of the network are listed at the end of the article

**Keywords:** Chagas disease, congenital, surveillance system, *Trypanosoma cruzi*, screening, public health, vertical transmission

## Abstract

**Background:**

Chagas disease is endemic in Latin America and affects 8 million people worldwide. In 2010, Catalonia introduced systematic public health surveillance to detect and treat congenital Chagas disease.

**Aim:**

The objective was to evaluate the health outcomes of the congenital Chagas disease screening programme during the first 6 years (2010–2015) after its introduction in Catalonia.

**Methods:**

In a surveillance system, we screened pregnant women and newborns and other children of positive mothers, and treated Chagas-positive newborns and children. Diagnosis was confirmed for pregnant women and children with two positive serological tests and for newborns with microhaematocrit and/or PCR at birth or serology at age 9 months.

**Results:**

From 2010 to 2015, the estimated screening coverage rate increased from 68.4% to 88.6%. In this period, 33,469 pregnant women were tested for *Trypanosoma cruzi* and 937 positive cases were diagnosed. The overall prevalence was 2.8 cases per 100 pregnancies per year (15.8 in Bolivian women). We followed 82.8% of newborns until serological testing at age 9–12 months and 28 were diagnosed with Chagas disease (congenital transmission rate: 4.17%). Of 518 siblings, 178 (34.3%) were tested and 14 (7.8%) were positive for *T. cruzi*. Having other children with Chagas disease and the heart clinical form of Chagas disease were maternal risk factors associated with congenital *T. cruzi* infection (p < 0.05).

**Conclusion:**

The increased screening coverage rate indicates consolidation of the programme in Catalonia. The rate of Chagas disease congenital transmission in Catalonia is in accordance with the range in non-endemic countries.

## Introduction

Chagas disease, a parasitic infection caused by the flagellated protozoan *Trypanosoma cruzi,* is endemic in Latin America [1]. It is found mainly in rural areas of Central and South America, except on the Caribbean islands, and coincides with the distribution of the vector that belongs to the family of triatomines and is responsible for transmission of the parasite to humans [2]. Other possible mechanisms of transmission are mother-to-child, blood transfusions, transplants of infected organs and tissues and ingestion of contaminated food [3]. There are an estimated 8 million people infected worldwide, of whom up to 30% may develop heart disease, with digestive or nervous system involvement in 10–20% [4-6].

Following migration from endemic areas to other countries, the epidemiological pattern of Chagas disease has changed in recent decades and new cases of congenital transmission and transmission by other mechanisms are detected in non-endemic countries [7]. The last decade (2000–2010) has seen an increase of people from endemic areas migrating to Europe [8]. In 2009, it was estimated that between 68,000 and 122,000 people from endemic countries living in Europe were infected, although the rate of underdiagnosis was 94–96% [8]. European prevalence rates in migrants from endemic areas differ greatly according to the country of origin, with an estimated prevalence rate of 4.2%, which rises to 18.1% in migrants from Bolivia [9].

Rates of congenital transmission in non-endemic countries are lower than those found in endemic countries [10] but the asymptomatic nature of the disease and the lack of knowledge about Chagas disease in non-endemic countries make it difficult to detect new cases [11,12]. Screening newborns of positive mothers is key to the early detection and treatment of possible cases in non-endemic countries [13].

Spain, for cultural reasons, is the European country that has received most migrants from Latin America [14]. Screening for *T. cruzi* in blood and tissue banks has been mandatory by Royal decree-law since 2005 [15] but legislation on the screening of congenital transmission is still lacking [11].

In Catalonia, estimates of people infected with *T. cruzi* in 2010 were between 10,000 and 20,000, with between 203 and 387 pregnant women affected and between seven and 16 children with congenital Chagas disease [16]. After confirming the cost-effectiveness of a screening programme for congenital Chagas disease [17] and following the recommendations of the World Health Organization (WHO) in non-endemic countries which had to take appropriate measures to prevent and control vertical transmission [11,18,19], the Deputy director of public health surveillance and response to emergencies of the Public Health Agency of Catalonia (PHAC) has since 2010 progressively introduced and coordinated a protocol to detect, treat and cure cases of congenital Chagas disease [20]. 

There is no common legislation on the control of the congenital transmission of Chagas disease in Europe, although there are regional initiatives for the early detection and treatment of cases according to WHO recommendations. Official programmes for the detection and treatment of congenital Chagas disease have been introduced in the Valencia (2008) [21], Catalonia (2010) [20] and Galicia (2014) [22] regions in Spain and in Toscana (2012) [23] in Italy. Other regions do not have an official protocol but act locally in hospitals [19,24-26].

The objective of this study was to analyse the epidemiological pattern of congenital Chagas disease in pregnant women from endemic areas and their children in the period from 2010 to 2015 in Catalonia and to evaluate the coverage of the screening programme.

## Methods

### Surveillance setting

Catalonia is an autonomous community in the north-east of Spain with more than 7.5 million inhabitants. In the study period (2010–2015), ca 450,000 people, 6% of the population, were born in countries where Chagas disease is endemic [27]. There are 45 public and 30 private maternity hospitals in Catalonia and 90% of births in Latin American women occur in public centres [28]. There are also 47 Sexual and Reproductive Health Care centres (*Centre d’Atenció a la Salut Sexual I Reproductiva* - ASSIR), distributed in 372 maternal assistance points, which form part of the network of public primary care centres. In addition, there are 27 microbiology laboratories able to perform diagnostic tests for Chagas disease [29].

### Screening of pregnant women, newborns and their siblings

We introduced a surveillance system to evaluate the impact of congenital Chagas disease in Catalonia. The target population were pregnant women from endemic countries (first or second generation) and pregnant women from other origins (including Spain) who have lived in a rural area of an endemic country for more than one month at any point in their lives.

**Figure 1:**
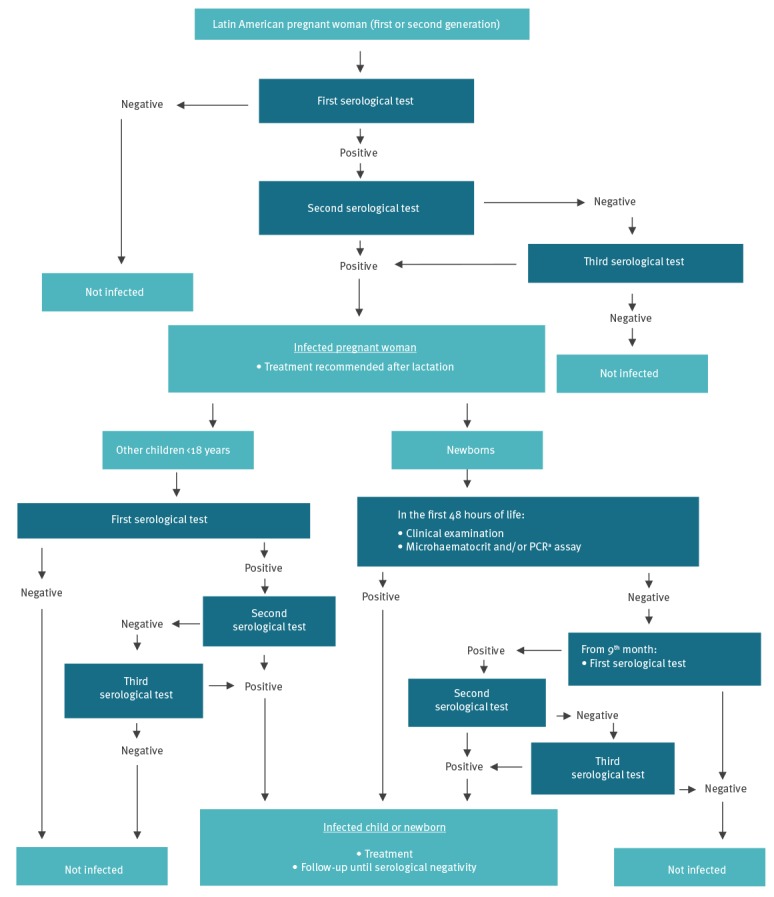
Congenital Chagas disease screening programme in Catalonia, 2010–2015

^a^ Many advances have been made in molecular biology and expert groups recommend PCR for the diagnosis in infants [28–30]. In case of positive PCR at birth, another PCR at age 1 month is necessary to confirm Chagas disease.

Serological screening is carried out during the first trimester of pregnancy, although tests done at any time during pregnancy, delivery or after birth are included in the programme (Figure 1) [20]. The tests used for screening are those recommended by the WHO [1]. Samples are collected at the ASSIR centre during pregnancy or in hospitals during or after delivery. If the first test is positive, a second test using a different antigen or serological technique is carried out. If the results between the two tests are discrepant, a third serological test, using a different technique, is carried out. All tests used in the programme follow the WHO recommendation [1] and laboratories choose recommended tests according to their own experience and supplier.

When the diagnosis is confirmed, it is recommended that pregnant women start treatment with trypanocidal drugs (benznidazole or nifurtimox) after birth and lactation, and before a possible new pregnancy. There is no risk of transmitting Chagas disease through breastfeeding.

Immediately after a birth to a mother diagnosed with Chagas disease, a clinical evaluation of the newborn is made in hospital to detect symptoms compatible with Chagas disease. The parasitological tests carried out during the first 48 h of life are the microhaematocrit and/or PCR [20]. If there is a positive PCR at birth, another PCR is carried out 4 weeks later to confirm the diagnosis. If any parasitological test is negative or tests cannot be carried out at birth, the infant is tested with a serological test after 9 months when maternal antibodies have waned. If this test is negative, the follow-up ends and the child is considered not infected; if the test is positive, a second serological test with a different technique is carried out. If the results of the two tests are discrepant, a third serological test, using a different technique, is carried out. If any microhaematocrit at birth, PCR at age 1 month or two serological tests after 9 months old are positive, *T. cruzi* infection is confirmed and antiparasitic treatment is administered.

The programme also includes other older children from positive mothers if they are living in Catalonia, using the same serological testing as for pregnant women. When two serological tests using different technique are positive, *T. cruzi* infection is confirmed and antiparasitic treatment is administered.

The screening and follow-up of pregnant women, newborns and siblings are included in the public health portfolio and are free of charge.

### Epidemiological surveillance

To implement the programme throughout the region, PHAC created the Working Group for Congenital Chagas disease in Catalonia, enrolling a large multidisciplinary group of Chagas disease experts who are responsible for the detection, notification and follow-up of positive pregnant women, newborns and siblings with positive mothers [16]: midwives, obstetricians, gynaecologists, paediatricians, microbiologists, specialists in infectious diseases and internal medicine, community health workers and epidemiologists.

Surveillance includes the mandatory notification of confirmed *T. cruzi* cases through the Microbiological Reporting System of Catalonia, a network of Catalonian laboratories that collects and reports pathogens of public health importance to the PHAC [30]. Reported cases are included in the Voluntary Registry of Chagas Disease Congenital Cases in Catalonia (VRCH). Sociodemographic, diagnostic and treatment data and epidemiological information about the mothers (years living in Catalonia, the clinical form of Chagas disease and previous treatments for Chagas disease) are voluntarily collected by the Working Group and included in the VRCH.

Laboratories report annually the number of pregnant women screened. To calculate the coverage of the screening of pregnant women, the denominator was estimated taking into account the number of births in women from endemic countries in the Register of Newborns (an official regional registry linked to each maternity hospital, public or private, which collects information on births in Catalonia, including the mothers’ country of origin [31]) and adding an estimation of pregnancies interrupted before giving birth (miscarriages and abortions) and women who moved away from Catalonia before childbirth as reported to the VRCH (13% of total pregnancies). Prevalence rates were calculated on pregnancies and not on pregnant women because the screening is repeated for each new pregnancy. To calculate the prevalence rates by country of origin we applied the distribution of births by maternal country of origin in the Register of Newborns to the total of pregnancies screened.

### Statistical analysis

All outcomes are shown in percentages and the annual percentage differences between 2010 and 2015 are shown as a relative change and evaluated using the Z score for two proportions of population.

Maternal epidemiological risk factors were evaluated between newborns with a definitive positive and negative diagnosis of Chagas disease. Continuous variables (age and years living in Catalonia) were transformed into categorical variables, choosing the mean as cut-off point. Statistical significance was established assuming an α error of 0.05. Differences between groups were analysed by simple logistic regression and the results are shown as p value and odds ratio (OR). Multiple logistic regression was used to calculate the adjusted OR (aOR) and variables with a p value < 0.20 in the crude analysis were entered in the model. To avoid the problem of quasi-complete separation, Firth logistic regression was used [32].

The analysis was performed using the Statistical Package for Social Sciences (SPSS v.25 for Windows).

### Ethical statement

The study was not submitted for approval by a research ethics committee because the activities described were conducted as part of the legislated mandate of the Health Department of Catalonia, the competent authority for the surveillance of communicable diseases according to Decree 203/2015 of 15 September, which created the epidemiological surveillance network of Catalonia [30]. All the activities studied formed part of public health surveillance and did not require informed consent.

## Results

Table 1 shows the overall results for screened pregnant women and follow-up in newborns and siblings.

**Table 1:** Screening of pregnant women and follow-up of siblings and newborns for Chagas disease, Catalonia, 2010–2015 (n = 40,084)

	Total		2010		2011		2012		2013		2014		2015		Difference 2010–2015	p value 2010 vs 2015
	n	%	n	%	n	%	n	%	n	%	n	%	n	%	%	
Pregnancies follow-up																
To be tested^a^	40,084		7,656		7,145		7,099		6,348		6,005		5,831		−23.8	NA
Tested	33,469	83.5	5,238	68.4	6,107	85.5	6,324	89.1	5,524	87.0	5,108	85.1	5,168	88.6	29.5	< 0.001
Positives	937	2.8	128	2.4	179	2.9	168	2.7	163	3.0	148	2.9	151	2.9	19.6	0.147
Lost before parturition^b^	38	4.1	8	6.3	8	4.5	11	6.5	3	1.8	3	2.0	5	3.3	−47.0	0.381
Miscarriages/abortions^b^	87	9.3	7	5.5	16	8.9	17	10.1	18	11.0	14	9.5	15	9.9	81.6	0.248
Gave birth^b^	812	86.7	113	88.3	155	86.6	140	83.3	142	87.1	131	88.5	131	86.8	−1.7	0.840
Other children follow-up																
Siblings to be tested	519		130		84		92		73		73		67		−48.5	NA
Missing information/not tested	341	65.7	121	93.1	42	50	44	47.8	49	67.1	52	71.2	33	49.3	−47.1	< 0.001
Tested	178	34.3	9	6.9	42	50.0	48	52.2	24	32.9	21	28.8	34	50.7	633.0	
Positives	14	7.9	2	22.2	3	7.1	2	4.2	2	8.3	2	9.5	3	8.8	−60.3	0.596
Negatives	164	92.1	7	77.8	39	92.9	46	95.8	22	91.7	19	90.5	31	91.2	17.2	
Newborns follow-up																
1. Parasitological control at birth																
Not tested	84	10.3	19	16.8	30	19.4	13	9.3	7	4.9	6	4.6	9	6.9	−59.1	0.026
Tested	728	89.7	94	83.2	125	80.6	127	90.7	135	95.1	125	95.4	122	93.1	12.0	
Tested by PCR	634	87.1	83	88.3	100	80.0	110	86.6	122	90.4	111	88.8	108	88.5	0.3	0.871
Tested by MH	419	57.6	73	77.7	59	47.2	69	54.3	75	55.6	73	58.4	70	57.4	−26.1	0.003
Positives	12	1.6	2	2.1	2	1.6	1	0.8	4	3.0	3	2.4	0	0	−100.0	0.367
Negatives	716	98.4	92	97.9	123	98.4	126	99.2	131	97.0	122	97.6	122	100	2.2	
2. Serological control at age 9–12 months																
To be tested	800		111		153		139		138		128		131		18.0	NA
Not tested	140	17.5	21	18.9	33	21.6	31	22.3	26	18.8	12	9.4	17	13.0	−31.4	0.276
Moved away from Catalonia	56	7.0	1	0.9	13	8.5	13	9.4	17	12.3	9	7.0	3	2.3	154.2	0.735
Failure to attend the medical visit	53	6.6	15	13.5	9	5.9	9	6.5	8	5.8	2	1.6	10	7.6	−43.5	0.199
Failure of surveillance circuit^c^	31	3.9	5	4.5	11	7.2	9	6.5	1	0.7	1	0.8	4	3.1	−32.2	0.800
Tested	660	82.5	90	81.1	120	78.4	108	77.7	112	81.2	116	90.6	114	87.0	7.3	0.276
Mean time to test (months)	10 ± 4		10 ± 3.5		10 ± 6		10±4		10 ± 4		10 ± 3		9 ± 4			
Positives	16	2.4	4	4.4	2	1.7	1	0.9	4	3.6	2	1.7	3	2.6	−40.8	0.750
Negatives	644	97.6	86	95.4	118	98.3	107	99.1	108	96.4	114	98.3	111	97.4	1.9	
3. Summary of testing																
Correctly tested	672	82.8	92	81.4	122	78.7	109	77.9	116	81.7	119	90.8	114	87	6.9	0.304
Positives	28	4.2	6	6.5	4	3.3	2	1.8	8	6.9	5	4.2	3	2.6	−59.6	0.318
Negatives	644	95.8	86	93.5	118	96.7	107	98.2	108	93.1	114	95.8	111	97.4	4.2	
Index																
Pregnant women screening coverage rate (%)	83.5		68.4		85.5		89.1		87.0		85.1		88.6		29.5	< 0.001
Prevalence rate of Chagas disease (%)	2.80		2.44		2.93		2.66		2.95		2.90		2.92		19.6	0.147
Congenital transmission rate (%)	4.17		6.52		3.28		1.83		6.90		4.20		2.63		−59.6	0.318

NA: not Applicable.

^a^ Number of births from the Newborns Registry plus an estimation (13%) of interrupted pregnancies and women who moved away before childbirth from the Voluntary Registry of Chagas disease congenital cases in Catalonia.

^b^ Among the women who tested positive.

^c^ Missing patient referral or paediatrician’s lack of awareness about Chagas disease.

### Screening of pregnant women

It was estimated that 40,084 pregnant women should have been tested in Catalonia between 2010 and 2015. Of these, 33,469 (83.5%) were actually screened, an annual mean of 5,578 tests (Table 1). No positive cases were detected in pregnant women who were second-generation migrants or travellers.

A total of 818 women were diagnosed with *T. cruzi* during pregnancy between 2010 and 2015: 707 (86%) became pregnant once, 103 twice (13%) and eight (1%) three times. In total, 937 pregnancies in positive women were followed between 2010 and 2015.

Screening coverage of pregnant women increased mainly between 2010 (68.4%) and 2011 (85.5%), when the logistics of the programme were introduced in all areas. The coverage gradually increased further until 2015 (88.6%) (p < 0.001 between 2010 and 2015).

**Figure 2:**
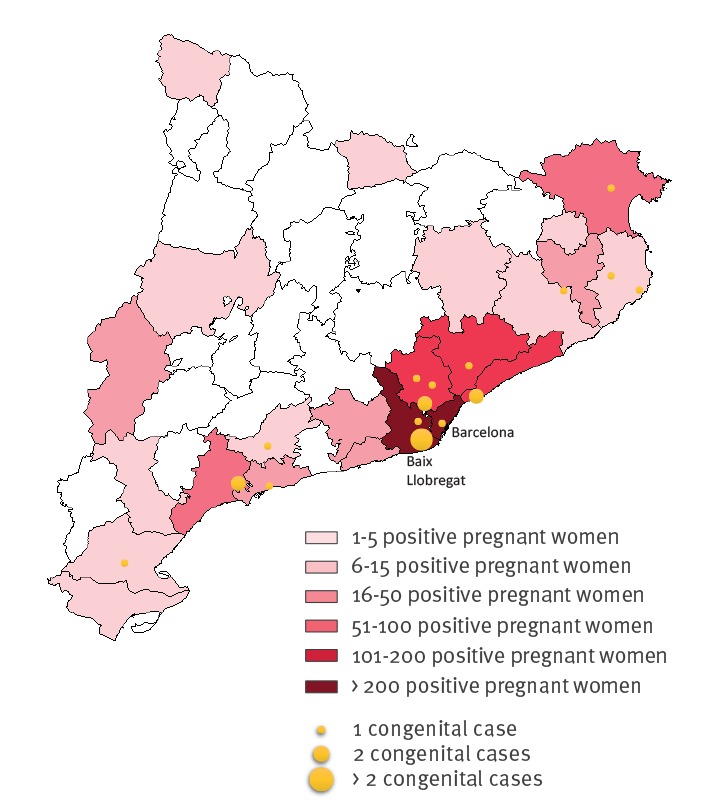
Geographical distribution of *Trypanosoma cruzi*-positive pregnant women and cases of congenital transmission, Catalonia, 2010–2015 (n = 818)

The highest density of Chagas-positive women was seen in the Barcelona health area (717 cases; 87.7%), especially in the Baix Llobregat (270 cases; 37.7%) and Barcelona (233 cases; 32.5%) areas (Figure 2).

**Figure 3:**
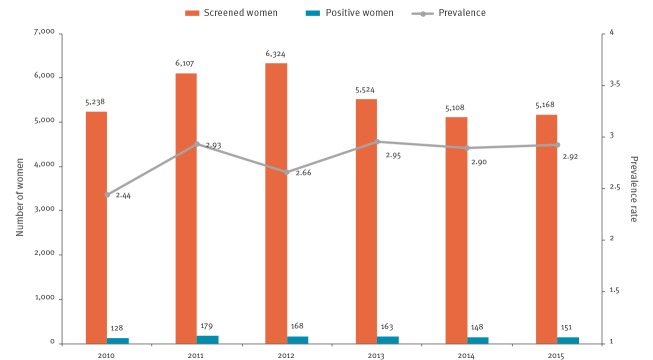
Annual number of screened women and *Trypanosoma cruzi*-positive pregnant women, Catalonia, 2010–2015 (n = 33,469)

During the study period, the prevalence rate was 2.8 positive cases per 100 pregnancies screened (Figure 3). The rates were highest in women from Bolivia (15.79), El Salvador (1.41) and Paraguay (1.24) (Table 2).

**Table 2:** Epidemiological characteristics, prevalence rates by endemic country of origin and maternal risk factors for congenital Chagas disease, Catalonia, 2010–2015 (n = 818)

Maternal risk factors	Positive pregnant women (n = 818)		Prevalence rates for country of origin^a^	Completed follow-up negative in newborns (n = 644)		Completed follow-up positive in newborns (n = 28)		Crude p value	Crude OR (CI)	Adjusted p value^b^	Adjusted OR (CI)^b^
	n	%		n	%	n	%				
Age											
< 33 years	389	47.6		305	47.4	17	60.7	0.166	1.71 (0.79–3.72)	0.693	1.30 (0.35–5.28)
≥ 33 years	429	52.4		339	51.6	11	39.3	Ref			
Previous treatment^c^											
Yes	115	26.0		107	29.9	2	8.3	Ref			
No	328	74.0		251	70.1	22	91.7	0.033	3.85 (1.02–14.49)	0.093	6.67 (0.78–876.89)
Country of birth^d^											
Bolivia	755	92.5	15.79	598	92.9	27	96.4	Ref			
Paraguay	20	2.5	1.24	17	2.6	1	3.6	0.801	1.30 (0.17–10.15)	NA	
Argentina	13	1.6	0.52	11	1.7	0	0	NA			
Ecuador	7	0.9	0.10	4	0.6	0	0	NA			
Honduras	6	0.7	0.26	4	0.6	0	0	NA			
Chile	5	0.6	0.50	1	0.2	0	0	NA			
El Salvador	4	0.5	1.41	3	0.5	0	0	NA			
Peru	4	0.5	0.11	4	0.6	0	0	NA			
Nicaragua	1	0.1	0.57	1	0.2	0	0	NA			
Colombia	1	0.1	0.02	1	0.2	0	0	NA			
Clinical form of Chagas disease^e^											
Indeterminate	524	94.1		432	94.9	23	88.5	Ref			
Heart	21	3.8		15	3.3	3	11.5	0.047	3.76 (1.02–13.90)	0.009	14.40 (2.11–87.67)
Digestive	9	1.6		8	1.8	0	0	NA			
Mixed	3	0.5		0	0	0	0	NA			
Siblings completing follow-up^f^											
Negative	131	92.3		150	97.4	4	40	Ref			
Positive	11	7.7		4	2.6	6	60	< 0.001	56.25 (11.26–280.9)	0.001	22.79 (3.75–161.54)
Years living in Catalonia^g^											
≤ 7 years	254	57.9		186	49.7	12	75	0.059	3.03 (0.96–9.57)	0.453	1.76 (0.42–10.05)
> 7 years	185	42.1		188	50.3	4	25	Ref			

CI: confidence interval; NA: Not Applicable; OR: odds ratio; Ref: reference value.

^a^ Number of positive newborns per 100 births, adjusted by pregnant women screening coverage rate.

^b^ Firth multiple logistic regression.

^c^ Unknown data: n = 375 (45.8%).

^d^ Unknown data: n = 2 (0.2%).

^e^ Unknown data: n = 261 (31.9%).

^f^ Mothers without other children and with untested other children: n = 676 (82.6%).

^g^ Unknown data: n = 379 (46.3%).

The mean age of positive women at pregnancy was 33 years, which increased from 32 years in 2010 to 34 years in 2015. Bolivian women represented 92.5% of the positive cases in whom the country of birth could be identified, followed by women from Paraguay (2.5%), Argentina (1.6%), Ecuador (0.9%), Honduras (0.7%), Chile (0.6%), El Salvador (0.5%), Peru (0.5%), Nicaragua (0.1%) and Colombia (0.1%). Almost half of the cases (47.6%) had arrived in Catalonia between 2005 and 2006, and the mean number of years from arrival to pregnancy was 7 years (Table 2).

The main clinical form of Chagas disease was indeterminate (94.1%). Women with heart clinical form represented 3.8% of cases, while digestive and mixed pathologies (heart and digestive) accounted for 1.6% and 0.5% of cases, respectively. Only 26% of pregnant women received treatment with benznidazole or nifurtimox before pregnancy. This percentage increased from 7.8% (7/90 cases with data about treatment) for pregnant women diagnosed in 2010 to 46.5% for those diagnosed in 2015 (33/71 cases with data about treatment) (p < 0.001).

Pregnancies were interrupted in 9.3% (n = 87) of pregnancies. More than half were miscarriages (65.5%), followed by abortions (23%) and cases where the reason for interruption was missing (11.5%), while 4.1% of pregnant women left Catalonia before childbirth, which meant that follow-up of the newborn was not possible.

There were 812 births from 937 pregnancies in 818 *T. cruzi*-positive women in Catalonian maternity hospitals (Table 1).

### Follow-up of siblings

For 674 of the 818 *T. cruzi*-positive women (82.4%) detected by the programme, it was possible to determine whether they had other children born before the current pregnancy and living in Catalonia, and 359 (53.3%) had at least one. The mean of other children per mother was 0.8. We identified 519 children for screening. In most cases, the children had not been tested or testing information not notified by the working group (341 cases; 65.7%), ranging from 93.1% in 2010 to 49.3% in 2015 (p < 0.001). Of the 178 children who were successfully screened and reported (34.3%), 14 were positive (7.9%) (Table 1). The median age of those 14 children was 10 years (range: 3–18 years) and 12 were male. Five of them were born in Catalonia between 2005 and 2008 but were not tested during the first year of life, while nine arrived in Catalonia during childhood. All 14 cases started treatment with benznidazole, but treatment was interrupted in two cases because of side effects such as neutropenia and toxicoderma and was not resumed, although the follow-up continued. In seven of the 14 cases, the follow-up was not completed with the required serological test. None of the seven children who continued the follow-up had negative serological tests after treatment, with a median follow-up of 4 years (range: 1–6 years) (Table 3).

**Table 3:** Positive *Trypanosoma cruzi* diagnostic tests, treatment and follow-up in newborns and their siblings, Catalonia, 2010–2015 (n = 42)

ID	Microhaematocrit	PCR	Serology	Country of birth	Age of arrival	Age at diagnosis (months)	Age at treatment start (months)	Symptoms compatible with Chagas disease	Adverse reactions to treatment	Completed treatment	Serological negativisation	Follow-up after treatment end (months)
Newborns												
1	+	+	NA	NA	NA	0	2	No	No	Yes	Yes	1
2	+	+	NA			0	0	Yes	Yes	No	Yes	9
3	+	+	NA			0	0	Yes	No	Yes	Yes	9
4	−	+	NA			0	1	No	No	Yes	Yes	1
5	+	NP	NA			0	0	Yes	No	Yes	Yes	6
6	NP	+	NA			0	0	No	No	Yes	Yes	21
7	−	+	NA			1	2	No	No	Yes	Yes	6
8	NP	+	NA			1	4	No	No	Yes	Yes	6
9	−	+	NA			1	1	No	No	Yes	No	34
10	+	NP	NA			1	3	No	No	Yes	Yes	3
11	−	+	NA			2	15	No	No	Yes	No	11
12	NP	+	NA			6	6	No	No	Yes	Yes	12
13	NP	−	+			9	9	No	No	Yes	Yes	5
14	−	NP	+			9	10	No	No	Yes	Lost^a^	NA
15	NP	NP	+			9	9	No	No	Yes	Yes	0
16	−	NP	+			9	11	No	No	Yes	Yes	9
17	NP	−	+			9	9	No	Yes	Yes	No	24
18	−	−	+			10	11	No	No	Yes	Lost^a^	NA
19	−	−	+			11	12	No	No	Yes	No	33
20	NP	NP	+			11	11	No	No	Yes	No	59
21	−	−	+			12	13	No	Yes	Yes	Yes	18
22	−	NP	+			12	12	Yes	No	No	Lost^a^	NA
23	−	−	+			13	12	No	No	Yes	No	35
24	NP	−	+			15	16	No	No	Yes	Yes	16
25	NP	NP	+			20	24	No	No	Yes	No	28
26	NP	NP	+			20	21	No	No	Yes	No	30
27	−	−	+			23	23	No	No	Yes	No	4
28	NP	−	+			27	28	No	Yes	Yes	No	27
Median (interquartile range)						9 (11)	9.5 (10.75)					11.5 (21.75)
Siblings												
1	NA	NA	+	Spain	NA	3	3	No	No	Yes	No	4
2			+	Spain	NA	4	5	No	No	Yes	No	4
3			+	Spain	NA	4	4	No	No	Yes	No	1
4			+	Bolivia	7	5	5	No	No	Yes	No	6
5			+	Spain	NA	7	7	No	Yes	No	Lost^a^	NA
6			+	Bolivia	1	7	8	No	Yes	No	Lost^a^	NA
7			+	Spain	NA	9	9	No	No	Yes	No	3
8			+	Bolivia	9	11	11	No	No	Yes	Lost^a^	NA
9			+	Bolivia	9	11	11	No	No	Yes	Lost^a^	NA
10			+	Bolivia	6	11	11	No	No	Yes	No	2
11			+	Bolivia	10	12	12	No	No	Yes	Lost^a^	NA
12			+	Bolivia	13	13	14	No	No	Yes	Lost^a^	NA
13			+	Bolivia	9	16	16	No	No	Yes	No	5
14			+	Bolivia	15	18	18	No	No	Yes	Lost^a^	NA
Median (interquartile range)						10 (7.5)	10 (7.5)					4 (3)

+: positive; −: negative; ID: identification number; NP: not performed; NA: not applicable.

^a^ Lost to follow-up before serological control.

### Follow-up of newborns

Of the 812 newborns, 728 (89.7%) were tested for *T. cruzi* parasite at birth. The most frequent tests were PCR (87.1%) and microhaematocrit (57.6%). In 84 of 812 cases (10.3%) the newborn was not tested at birth (16.8% in 2010 and 6.9% in 2015; p = 0.029). Testing after age 9 months was carried out in 672 of 812 newborns (82.8%). Of these, 95.8% (n = 644) tested negative. The median age at screening was 10 months (Table 1).

A total of 140 newborns (17.5%) did not complete the follow-up. The main reason was the departure of the family from Catalonia before the newborn was 9 months old (7.0%), followed by failure to attend the medical visit (6.6%) and failure of the surveillance circuit (3.9%).

Twenty-eight cases were diagnosed with *T. cruzi* infection acquired through congenital transmission (4.2%). In 27 cases, the mother was from Bolivia and in one case from Paraguay. Twelve infants were diagnosed by parasitological tests before age 9 months and 16 infants with serological tests after age 9 months (Table 3). In four of 28 cases, the newborn presented symptoms compatible with Chagas disease, including splenomegaly (3/4), hepatomegaly (3/4) and jaundice (3/4).

All 28 positive cases were treated with benznidazole. Treatment was suspended because of failure to attend follow-up visits in one case and because of an adverse reaction in one case. Overall, four of 28 newborns had adverse reactions, including increased transaminases (n = 1), pancytopenia (n = 1), cessation of weight gain (n = 1) and anorexia (n = 1). Serology after treatment was negative in 15 cases, with a mean time between treatment end and serology of 8.1 months (range: 0–21 months). Two newborns treated before age 12 months did not become seronegative: the first was diagnosed by PCR 1 month after birth and remained positive 1 year after treatment. Treatment was repeated 4 years later and the subsequent PCR was negative, but serological testing was not carried out. The second child had a negative PCR at birth, but positive PCR and serology at 9 months. Treatment was stopped after 10 days because of pancytopenia and was resumed 2 months later. Two years later serology remained positive.

Recovery rates were 89% for newborns treated before 6 months of age, 80% for those treated between 6 and 12 months of age and 20% after 12 months of age. Taking the serological diagnosis after 9 months as the gold standard, the sensitivity of the microhaematocrit and PCR was 29.4% and 52.6%, respectively, and the specificity 100% and 99.2% (in four cases, PCR was positive at birth but negative after 1 month).

### Analysis of maternal risk factors

In an analysis of maternal risk factors for vertical transmission of the infection, we saw significant differences between positive and negative siblings (aOR = 22.79; 95% confidence interval (CI): 3.75–161.54) and between heart and indeterminate clinical forms (aOR = 14.4; 95% CI: 2.11–87.67) (Table 2). Differences between untreated and treated mothers showed crude statistical significance (p = 0.033) but significance was lost after adjusting for multivariate logistic regression (aOR = 6.67; 95% CI: 0.78–876.89). Other risk factors analysed, such as the mother’s age, country of origin or time living in Catalonia (≤ 7 years) had no significant influence on the likelihood of vertical transmission.

## Discussion

The congenital Chagas disease prevention and control programme in Catalonia is one of few screening programmes for the control of congenital Chagas disease launched by public health authorities in a non-endemic region [9].The observed prevalence of Chagas disease (2.8 cases/100 pregnancies) was similar to that found in other studies in pregnant women in Catalonia [33, 34]. The prevalence in Bolivian pregnant women was lower (15.8%) than in a similar programme in Bolivia (23.3%) [35]. Studies in other regions in Spain show higher prevalence rates in Valencia (34.1%) [36] and Vizcaya (22%) [37] but lower rates in Madrid (11.4%) [38] and Almeria (12.5%) [39]. Other non-endemic countries show lower rates in Bolivian pregnant women living in Italy (8.7%) [25] and Switzerland (8.8%) [24]. These differences may be due, in part, to methodological differences in estimating the rates. The prevalence rates observed in our programme in women from other endemic countries such as Paraguay, Argentina, Ecuador, Honduras, Chile, el Salvador, Peru, Nicaragua and Colombia (range: 0.02–1.41), were much lower than those detected in other Spanish studies (range: 0.2–7.4) [36,40] or in studies from the endemic countries themselves (range: 3.2–12.7) [41-45].

The rate of congenital transmission in Catalonia (4.17%) was within the range detected in endemic (range: 1.7–5) [35,46-50] and non-endemic countries (range: 0–7.3) [25,33,34,36-38].

The estimated screening coverage rate in pregnant women was 83.5%, which is lower than the rate found in Valencia (94.5%) [36]. This may be due, in part, to the greater centralisation and smaller number of centres included in the Valencia programme (three maternity hospitals) compared with Catalonia (45 public maternity hospitals and 372 primary health centres with midwife care, including all public health centres).

Screening of the newborns’ siblings is widely neglected in gestational screening programmes and there are few studies of this subgroup [51-53]. A prevalence study conducted in Catalonia in children younger than 18 years with a Chagas disease-positive mother [53] found a slightly higher rate (10.9%) than ours (7.3%), and a clinical study of children in Catalonia and Switzerland identified a higher percentage of adverse effects during treatment (36%) than our programme (14.3%) and a recovery rate at age 2 years of 17.2%, compared with 0% in our programme [51]. Although the screening of other children improved significantly between 2010 (6.9%) and 2015 (50.7%), the high percentage of missing cases (352 cases, 66.4%), and the missing follow-up in positive cases (50%) demonstrate a lack of a well-established notification and follow-up circuit for this subgroup.

Parasitological testing at birth improved significantly between 2010 (83.2%) and 2015 (93.1%). PCR was used more than the microhaematocrit (87.1% and 57.6%, respectively), and the microhaematocrit was less frequent in 2015 than in 2010 (57.4% vs 77.7%). This confirms the greater practicality of PCR in our region. Even if PCR is widely accepted for the early diagnosis of Chagas disease [54-56], false positive (four cases) and false negative results (nine cases) indicate that a standardised PCR technique with higher sensitivity is required [57]. Currently, it is still necessary to wait until age 1 month to validate the diagnosis by PCR or to perform a serological test after 9 months for PCR-negative cases [58].

We detected some delay between diagnosis and start of treatment for positive newborns. There are several possible explanations: the presence of other pathologies that require other incompatible treatments, difficulty in obtaining the medication (there was a significant lack of supply of Benznidazole a few years ago) or a decision made by the patient's family. 

The recovery rates observed in treated newborns are provisional data because newborns with positive serology will be followed until serology is negative and the results could therefore change in the future. Sometimes problems with treatment compliance or adverse reactions can affect seronegativisation in post-treatment follow-up. However, although our results are based on very few cases, the current results suggest that it is very important to detect the infection before age 12 months to achieve a probability of cure of more than 80%. Schijman et al. found a 100% recovery rate when treatment is started before age 6 months compared with 88.9% in our study [59]. 

With respect to maternal epidemiological risk factors for congenital transmission, we found three studies that showed an increased risk of congenital transmission in untreated women [60-62]. In our study, women untreated before pregnancy had an almost sevenfold greater probability of congenital infection but the adjusted significance was weak (p = 0.093). Having the heart clinical form of Chagas disease rather than the indeterminate clinical form and having other infected children increased the risk of congenital transmission 14 and 23 times, respectively. These findings demonstrate the importance of recommending treatment of women of childbearing age before a new pregnancy, especially in those who already have infected children or those with the heart clinical form of the disease.

Other studies in Catalonia found a higher proportion of the digestive clinical form (up to 21% vs 1.6%) [5,6]. Our results may be an underestimate because infected women diagnosed during pregnancy could not undergo specific radiological tests to detect possible digestive disorders.

The main challenge of our programme was to calculate the coverage of screening for pregnant women and the prevalence rate by country of origin, because the protocol did not plan for quantifying the target population and collecting epidemiological information on pregnant women with negative results. To solve this limitation, we used the Register of Newborns as a source. It will be necessary to involve the ASSIR centres in reporting all cases, negative or positive, or create an improved data collection system to provide this information. Another limitation of the programme were the 10.5% missing numbers in the follow-up at age 9–12 months owing to failures in the follow-up circuit such as a lack of awareness about Chagas disease among paediatricians and patients, or a missing patient referral. The percentage lost to follow-up was smaller in 2013 (6.5%) and 2014 (2.4%) because of a specific community health action to redirect lost cases [63]. It is therefore necessary to improve primary healthcare circuits to control the newborns and other children of positive mothers and to add community health actions to the surveillance of congenital Chagas disease.

## Conclusion

The results of the congenital Chagas disease programme in Catalonia show that systematic control of the congenital transmission of Chagas disease by an integrated public health surveillance system is possible in a non-endemic region and the increase in the estimated screening coverage rate indicates its consolidation in Catalonia.

Prevalence and congenital transmission rates were within the ranges detected in other studies conducted in non-endemic settings. Having previous children with Chagas disease and presenting the heart clinical disease form of the disease were risk factors for the congenital transmission of *T. cruzi*. Treatment of women of childbearing age with these characteristics is recommended in order to improve the treatment of Chagas disease in non-endemic countries.

## Members of the Working Group of Congenital Chagas disease in Catalonia

Alba Cebollero. CLILAB Diagnòstics, Consorci del Laboratori Intercomarcal.

Alicia Carrascon. ASSIR Izquierdo (Barcelona ciudad), Institut Català de la Salut (ICS).

Ana Moreira. Hospital Sant Joan de Déu de Martorell.

Ana Requena-Mendez. ISGlobal, Hospital Clínic de Barcelona, Universitat de Barcelona.

Ana Torres Soto. Hospital Residència Sant Camil.

Andrea Martín. Hospital Universitari Vall d’Hebron.

Anna Ballester. Corporació de Salut del Maresme i la Selva.

Anna Bragulat. Hospital de la Cerdanya.

Anna Soligó. ASSIR Consorci Sanitari de Terrassa.

Anna Vilamala Bastarras. Consorci Hospitalari de Vic.

Antoni Payà. Hospital del Mar.

Antoni Soriano-Arandes. Hospital Universitari Vall d’Hebron.

Antoni Sorni. Hospital Verge de la Cinta de Tortosa.

Antonia Calvo. Hospital Clínic de Barcelona.

Antonio Mur. Hospital del Mar.

Assumpta Colomer. Consorci Hospitalari de Vic.

Begoña Carral. ASSIR Baix Llobregat Litoral, ICS.

Begoña Treviño. Unitat de Salut Internacional Drassanes, Hospital Universitari Vall d’Hebron.

Borja Guarch. Hospital Universitari Dr Josep Trueta de Girona.

Carles Orta. Hospital Comarcal de l'Alt Penedès.

Carlos Rodrigo. Hospital Universitari Germans Trias i Pujol.

Carme Gallés. Corporació de Salut del Maresme i la Selva.

Carme Garrido. Hospital de la Santa Creu i Sant Pau.

Carme Molina. Hospital Joan XXIII de Tarragona.

Carme Mora. Hospital de Figueres.

Carmen Muñoz. Hospital de la Santa Creu i Sant Pau.

Carmina Martí. Hospital General de Granollers.

Carmina Sanjosé. CLILAB Diagnòstics, Consorcio del Laboratorio Intercomarcal.

Celia Guardia. Laboratori Barcelonès Nord i Vallés Oriental.

Cristina Cortes. Hospital General de l'Hospitalet.

Cristina Gutierrez. Hospital Universitari Joan XXIII de Tarragona.

Cristina Martinez. ASSIR Barcelona Ciutat, ICS.

Cristina Riera. Facultat de Farmàcia. Universitat de Barcelona.

Cristina Soler. Hospital de Santa Caterina.

Dolors Gonzalez. CAP Rambla Nova. ICS Camp de Tarragona.

Dolors Guix. ASSIR Granollers, ICS.

Eduard Solé Mir. Hospital Universitari Arnau de Vilanova, IRBLleida.

Eduardo Padilla. Laboratori de Referència de Catalunya.

Elena Sulleiro. Hospital Universitari Vall d’Hebron.

Elisa Llurba. Hospital de la Santa Creu i Sant Pau.

Elisabeth Del Amo. ASSIR Litoral (Barcelona ciudad), ICS.

Elisenda Moliner. Hospital de la Santa Creu i Sant Pau.

Emma Padilla Esteba. Catlab-Centre Analítiques Terrassa, AIE.

Engràcia Coll. ASSIR Hospital Mutua Terrassa, ICS.

Enrique Rodriguez. Hospital General de l'Hospitalet.

Enrnesto Monaco. Hospital Residencia Sant Camil.

Ester Muñoz. Hospital de la Cerdanya.

Esther Freixas. ASSIR l’Hospitalet, ICS.

Eva Dopico. Laboratori Clínic L’Hospitalet.

Eva Sardá. ASSIR Muntanya (Barcelona ciudad), ICS.

Federic Ballester. Hospital Universitari Sant Joan de Reus.

Federic Gomez. Hospital Joan XXIII de Tarragona.

Ferran Barranco. Hospital de la Cerdanya.

Francesc Ripoll. Hospital Santa Caterina.

Francesc-Josep Fargas. ASSIR Camp de Tarragona, ICS.

Gemma Falguera. ASSIR Direcció d’Atenció Primària Metropolitana Nord, ICS.

Gema Fernández-Rivas. Microbiology Department, Clinical Laboratory North Metropolitan Area, Hospital Universitari Germans Trias i Pujol.

Gemma Ginovart. Hospital de la Santa Creu i Sant Pau.

Gemma Navarro. Hospital Universitari Parc Taulí.

Graciano García-Pardo. Hospital Joan XXIII de Tarragona.

Gustavo Lorenzo. Hospital de Mollet del Vallès.

Hakima Ouaarab. Unitat de Salut Internacional Drassanes, Hospital Universitari Vall d’Hebron.

Imma Caubet. Espitau Vall d’Aran.

Isabel Claveria. Unitat de Salut Internacional Drassanes, Hospital Universitari Vall d’Hebron

Isabel Sanfeliu. Hospital Universitari Parc Taulí.

Israel Molina. Hospital Universitari Vall d’Hebron.

Jesus Blanch. Hospital Residencia Sant Camil.

Joan Agullo Martí. Hospital de Palamós.

Joan Farré. Hospital Universitari Arnau Vilanova de Lleida.

Joaquim Gascón. ISGlobal, Hospital Clínic, Universitat de Barcelona.

Jordi Costa. Hospital Universitari Parc Taulí.

Jordi Gomez i Prat. Unitat de Salut Internacional Drassanes, Hospital Universitari Vall d’Hebron.

Jose Fulquet. Hospital de Figueres.

Josefa Perez Jove. Catlab-Centre Analítiques Terrassa, AIE.

Josep Armengol. Hospital de la Santa Creu i Sant Pau.

Juan Carlos Riera. ASSIR Gironès Pla de l'Estany, ICS.

Judith Villar. Parc de Salut Mar.

Laia Sanchez Torrent. Hospital General del Parc Sanitari Sant Joan de Déu (Sant Boi de Llobregat).

Leonardo De La Torre. ISGlobal, Hospital Clínic de Barcelona.

Lluis Delgado. Hospital Comarcal de l’Alt Penedès.

Lluis Valerio. Unitat de Salut Internacional Metropolitana Nord, ICS.

Lourdes Montsant. Hospital de la Cerdanya.

Luca Basile. Agència de Salut Pública de Catalunya.

Luis Mayol. Hospital Universitario de Girona Doctor Josep Trueta

M. Eugenia Valls. ISGlobal, Hospital Clínic de Barcelona.

MªAngels Vives. Hospital de Terrassa.

MªGoretti Sauca. Hospital de Mataró.

Maite Coll. Hospital General de Granollers.

MªJesus Pinazo. ISGlobal, Hospital Clínic de Barcelona.

MªJosé Ferri. Hospital Universitari Dr Josep Trueta de Girona.

MªJosé Vidal. Agència de Salut Pública de Catalunya.

MªLuz Villegas. Hospital General de l'Hospitalet.

Manuel Andres Samper Anquela. PIUS Hospital de Valls.

Manuel Monsonis. Hospital Maternoinfantil Sant Joan de Déu.

MªPilar Blasco. ASSIR Santa Coloma de Gramenet, ICS.

Mar Olga Pérez-Moreno. Hospital Verge de la Cinta de Tortosa.

Margarida Curriu Sabates. Hospital Sant Bernabé.

Maria Mèndez. Hospital Germans Trias i Pujol

Maria Navarro. Consorci Hospitalari de Vic.

Marisa Urcola. Hospital de Santa Caterina.

Marta Lora. Hospital Universitari Dr Josep Trueta de Girona.

Marta Navarro. Hospital Universitari Parc Taulí.

Merce Almirall. Hospital Universitari Arnau Vilanova de Lleida.

Mercè Arasa. ASSIR Terres de l'Ebre, ICS.

Meritxell Vidal Alaball. ASSIR Baix Empordà, Hospital de Palamós.

Mireia Carulla. Hospital de Sant Pau i Santa Tecla.

Mireia Jané. Agència de Salut Pública de Catalunya.

Monica Ribell-Bachs. Hospital General de Granollers.

Montse Abella. ASSIR Sabadell, ICS.

Montserrat Gallego. ISGlobal, Hospital Clínic de Barcelona.

Neus Prat. ASSIR Baix Llobregat, ICS.

Neus Rius. Hospital Universitario Sant Joan de Reus

Nuria Prim Bosh. Laboratori de Referència de Catalunya.

Pablo Garcia. Hospital Comarcal Móra d’Ebre.

Paloma Araujo. Hospital de Terrassa.

Pepa Solé. Hospital de Mataró.

Pilar Ciruela. Agència de Salut Pública de Catalunya.

Pilar Villalobos. Hospital de Figueres.

Rosa Almirall. ASSIR Izquierdo (Barcelona ciudad), ICS.

Rosa Diaz. ASSIR Mataró, ICS.

Rosa Puigarnau. Hospital Mútua Terrassa.

Rosa Serra. ASSIR Gironès Pla de l'Estany.

Roser Diez. Hospital de Mataró.

Sara Torrent Bosch. Hospital Universitario Dr Josep Trueta de Girona.

Silvia Franch. Hospital de Sant Pau i Santa Tecla.

Sonia Vega. Hospital de Figueres.

Susanna Garcia Mani. ASSIR Baix Llobregat Nord, ICS.

Teresa Juncosa. Hospital Maternoinfantil Sant Joan de Déu.

Valentí Pineda. Hospital Parc Taulí de Sabadell

Vicky Fumado. Hospital Maternoinfantil Sant Joan de Déu.

Xavier Urquizu. Hospital Mutua de Terrassa.
